# Cytotoxic Polyhydroxylated Oleanane Triterpenoids from *Cissampelos pareira* var. *hirsuta*

**DOI:** 10.3390/molecules27041183

**Published:** 2022-02-10

**Authors:** Yanjun Sun, Ruyi Pan, Haojie Chen, Chen Zhao, Ruijie Han, Meng Li, Guimin Xue, Hui Chen, Kun Du, Junmin Wang, Weisheng Feng

**Affiliations:** 1 Collaborative Innovation Center for Respiratory Disease Diagnosis and Treatment & Chinese Medicine, Development of Henan Province, Henan University of Chinese Medicine, Zhengzhou 450046, China; 18837138537@163.com (R.P.); CHj3928@126.com (H.C.); 15136725586@163.com (C.Z.); 18638221936@163.com (R.H.); limeng31716@163.com (M.L.); xueguimin123@126.com (G.X.); chenhuiyxy@hactcm.edu.cn (H.C.); qqninenine@hotmail.com (K.D.); wjmhnzz@163.com (J.W.); 2 School of Pharmacy, Henan University of Chinese Medicine, Zhengzhou 450046, China; 3 Henan Research Center for Special Processing Technology of Chinese Medicine, Zhengzhou 450046, China

**Keywords:** *Cissampelos pareira* var. *hirsute*, polyhydroxylated triterpenoids, oleanane, cytotoxic

## Abstract

Three new polyhydroxylated oleanane triterpenoids, cissatriterpenoid A−C (**1**−**3**), along with one known analogue (**4**), were isolated from the whole plant of *Cissampelos pareira* var. *hirsuta*. Their chemical structures were elucidated by extensive spectroscopic data (IR, HR-ESI-MS, ^1^H-NMR, ^13^C-NMR, DEPT, ^1^H-^1^H COSY, HSQC, HMBC, NOESY) and the microhydrolysis method. The isolation of compounds **1**–**4** represents the first report of polyhydroxylated oleanane triterpenoids from the family Menispermaceae. All isolated compounds were evaluated for their cytotoxicity against five human cancer cell lines, and the inhibitory activity against NO release in LPS-induced RAW 264.7 cells. Compound **3** showed the most potent cytotoxic activities against the A549, SMMC-7721, MCF-7, and SW480 cell lines, with IC_50_ values of 17.55, 34.74, 19.77, and 30.39 μM, respectively, whereas three remaining ones were found to be inactive. The preliminary structure–activity relationship analysis indicated that the γ-lactone ring at C-22 and C-29, and the olefinic bond at C-12 and C-13 were structurally required for the cytotoxicity of polyhydroxylated oleanane triterpenoids against these four cell lines. Based on lipid-water partition coefficients, compound **3** is less lipophilic than **1** and **4**, which agrees with their cytotoxic activities. This confirms the potential of *C. pareira* var. *hirsuta* in the tumor treatment.

## 1. Introduction

So far, cancer has become the most common disease threatening human health and life worldwide. Natural products, characteristic of low toxicity and high efficiency have played an irreplaceable role in cancer chemotherapy and chemoprevention in the last half-century. Over 60% of clinical anticancer drugs come from natural products, such as paclitaxel, etoposide, camptothecin, and vincristine, etc [1]. Oleanane triterpenoids are phyrophosphate oligomers of six isopentenyl structure units. They possess extensive and important biological activities, such as cardio-, hepato-, and gastro-protective, anti-inflammatory, antiviral, antidiabetic, antimicrobial, antiparasitic, analgesic and wound-healing effects, as well as inducing apoptosis in cancer cells [2]. Among these biological properties, their anti-cancer effects are most attractive as they are confirmed in various pharmacological models in vitro and in vivo [3]. Oleanane triterpenoids exhibit cytotoxic effects in many cancer cell lines, such as oral, esophageal, liver, brain, colorectal, ovary, breast, lung, skin cancers, and leukemia [4]. They regulate multiple cellular signaling pathways, including nuclear factor-KB, AKT, signal transducer and activator of transcription 3, mammalian target of rapamycin, caspases, intercellular adhesion molecule 1, vascular endothelial growth factor, and poly (ADP-ribose) polymerase in cancer cells [5]. As promising anti-cancer agents, they inhibit the viability and proliferation of human cancer cells, prevent their migration and metastasis, and induce their apoptosis [6]. So, oleanane triterpenoids will be a significant source of new anticancer-drug development.

**Figure 1:**
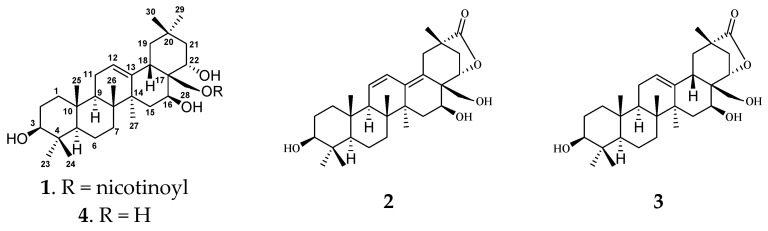
Chemical structures of compounds **1**–**4**.

*Cissampelos pareira* L. var. *hirsuta* (Buch. ex DC.) Forman is a perennial climbing shrub of Menispermaceae family, which is widely distributed in the Southwest of China, India. Its whole plant has been commonly used for the treatment of stomachache, cardiodynia, fever, skin disease, and swelling pain, etc [7]. However, very few researches on its chemical compositions have been reported. Previous phytochemical investigation of the genus *Cissampelos* showed the presence of alkaloids [8], flavonoids [9], terpenes [10], and pregnane glycosides [11]. Our previous chemical and pharmacological investigations on *C. pareira* var. *hirsuta* revealed the presence of polyhydroxylated pregnane glycosides, and their cytotoxic properties [11]. As our ongoing research for potential cytotoxic natural products from traditional Chinese medicines, three new polyhydroxylated oleanane triterpenoids, cissatriterpenoid A−C (**1**−**3**), and one known analogue (**4**), were obtained from the dried whole plant of *C. pareira* var. *hirsuta* (Figure 1). Detailed isolation, structure elucidation, and cytotoxicity, and NO inhibitory activity of those isolates are reported herein.

## 2. Results and Discussion

The 95% EtOH extract of the whole plant of *C. pareira* var. *hirsuta* was partitioned between petroleum ether (PE), CH_2_Cl_2_, n-BuOH and water, respectively. The CH_2_Cl_2_ layer was fractionated and purified by repeated column chromatography, allowing for the isolation of three new polyhydroxylated oleanane triterpenoids, cissatriterpenoid A−C (**1**−**3**), along with one known analogue (**4**). By comparing their physical and spectroscopic data with the literature values, the known metabolite was identified as chichipegenin (**4**) [12].

**Figure 2:**
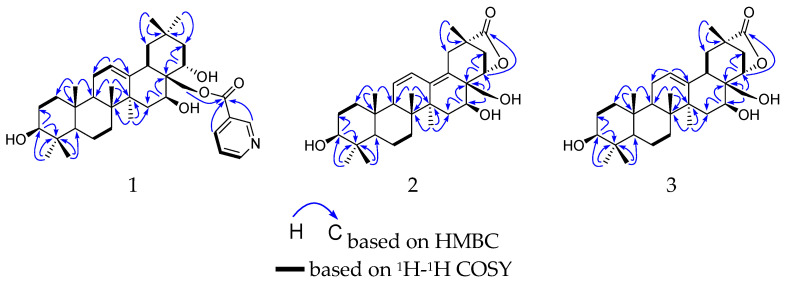
Key ^1^H-^1^H COSY and HMBC correlations of compounds **1**–**3**.

Compound **1** was obtained as a white amorphous solid and possessed a molecular formula C_36_H_53_NO_5_ with eleven degrees of unsaturation, as revealed from its HR-ESI-MS analysis (*m*/*z* 618.3559 [M + K]^+^, calcd 618.3561). The IR spectrum displayed the presence of ester carbonyl (1726 cm^−1^), olefinic (1593 cm^−1^), and hydroxy (3341 cm^−1^) groups. The ^1^H NMR spectrum (Table 1, Figure S1) showed one oxymethylene group *δ*_H_ 4.68 (1H, d, *J* = 11.0 Hz, H-28) and 4.55 (1H, d, J = 11.0 Hz, H-28), three oxymethine resonances *δ*_H_ 3.14 (1H, dd, *J* = 11.3, 4.7 Hz, H-3), 4.77 (1H, dd, *J* = 11.7, 5.4 Hz, H-16), and 4.32 (1H, dd, *J* = 12.6, 4.7 Hz, H-22), along with one nicotinoyl group *δ*_H_ 9.14 (1H, brs, H-3′), 8.40 (1H, dt, *J* = 8.0, 1.9 Hz, H-5′), 7.59 (1H, dd, *J* = 8.0, 5.1 Hz, H-6′), and 8.76 (1H, d, *J* = 3.9 Hz, H-7′) [13]. Seven tertiary methyl groups *δ*_H_ 0.98 (6H, s, Me-23, Me-29), 0.78 (3H, s, Me-24), 0.96 (3H, s, Me-25), 1.08 (3H, s, Me-26), 1.30 (3H, s, Me-27), and 1.03 (3H, s, Me-30), together with one olefinic proton *δ*_H_ 5.27 (1H, t, *J* = 3.4 Hz, H-12), were evidence for a characteristic olean-12-ene pentacyclic triterpenoid skeleton [14]. The ^13^C-NMR and DEPT spectra (Table 1, Figures S2 and S3) provided 36 carbon signals, including seven methyls, nine methylenes [one oxygenated *δ*_C_ 64.1 (C-28)], eleven methines [five olefinic *δ*_C_ 125.5 (C-12), 150.9 (C-3’), 138.8 (C-5’), 125.4 (C-6’), 154.2 (C-7’), and three oxygenated *δ*_C_ 79.6 (C-3), 67.5 (C-16), and 70.9 (C-22)], and nine quaternary carbons [two olefinic *δ*_C_ 142.3 (C-13), 128.1 (C-4’), an ester carbonyl *δ*_C_ 166.2]. The abovementioned data suggested that compound **1** is a polyhydroxylated oleanane triterpenoid derivative with the same 3,16,22,28-tetrahydroxyolean-12-ene skeleton as chichipegenin (**4**) [12]. The three OH groups were linked to C-3 (*δ*_C_ 79.6), C-16 (*δ*_C_ 67.5), and C-22 (*δ*_C_ 70.9), respectively, due to the HMBC correlations (Figure 2 and Figure S6) of H-3 (*δ*_H_ 3.14) with C-2 (*δ*_C_ 27.8), C-4 (*δ*_C_ 39.9), C-24 (*δ*_C_ 16.3), of H-16 (*δ*_H_ 4.77) with C-15 (*δ*_C_ 36.3), C-17 (*δ*_C_ 44.9), C-28 (*δ*_C_ 64.1), and of H-22 (*δ*_H_ 4.32) with C-17 (*δ*_C_ 44.9), C-21 (*δ*_C_ 44.1), C-28 (*δ*_C_ 64.1). The HMBC correlation between H-28 (*δ*_H_ 4.55, 4.68) and C-1’ (*δ*_C_ 166.2), indicated that 28-OH was esterified by nicotinic acid.

**Figure 3:**
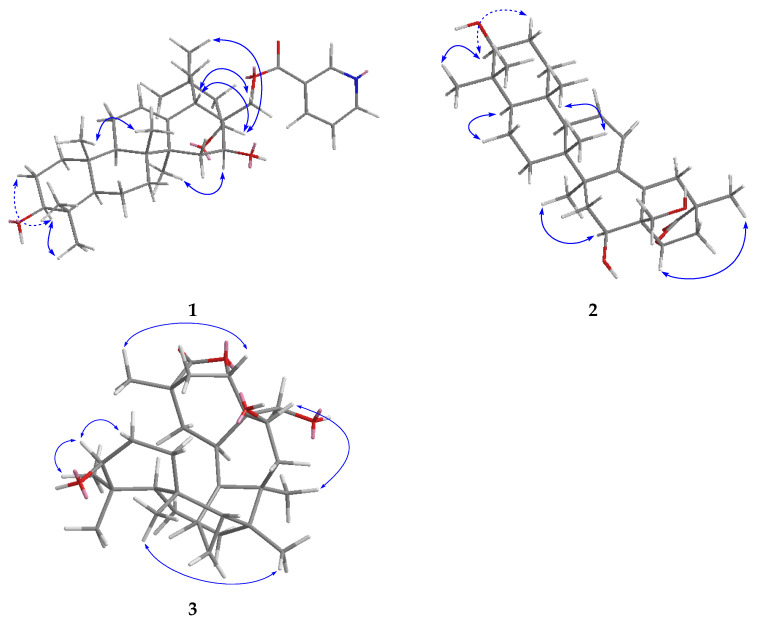
The key NOESY correlations of compounds **1**–**3**.

The relative configuration of the ring substituents of compound **1** was determined by analyzing the vicinal coupling constants of the key protons and NOESY spectrum (Figure 3 and Figure S7). The proton spin-coupling constants of J_H-2*β*/H-3_ (11.3 Hz), J_H-5/H6*β*_ (12.8 Hz), J_H-9/H-11*β*_ (11.9 Hz), J_H-18/H-19*α*_ (14.0 Hz), and J_H-21*α*/H-22_ (12.6 Hz) indicated that H-3, H-5, and H-9 were cofacial and were assigned as *α*-orientation [14], and this also supported the *β*-orientation of 3-OH. The NOESY correlations (Figure 3) from H-16 to H-27, from H-22 to H-30 and H-18 indicated that *β*- and *α*-orientations were assigned for 16-OH and 22-OH, respectively. The absolute configuration was the same as chichipegenin [12], which was determined by a microhydrolysis method and a comparison of their specific rotatory values. On the basis of the above information, compound **1** was established as 28-*O*-nicotinoyl-3*β*,16*β*,22*α*-trihydroxyolean-12-ene, and named cissatriterpenoid A.

Compound **2** was obtained as a white amorphous solid and possessed a molecular formula C_30_H_44_O_5_ with nine degrees of unsaturation, as revealed from its HR-ESI-MS analysis (*m*/*z* 507.3079 [M + Na]+, calcd 507.3086). The IR spectrum displayed the presence of carbonyl (1766 cm^−1^) and hydroxy (3383 cm^−1^) groups. Its ^1^H and ^13^C NMR spectra (Table 1, Figures S8 and S9) were similar to those of compound **1**, with the obvious difference being the absence of a nicotinoyl group in **2**. Furthermore, four olefinic carbons δ_C_ 124.9, 129.9, 141.7, 126.8 and one carbonyl group δ_C_ 182.9 in **2** were observed in contrast to the two olefinic carbons δ_C_ 125.5, 142.3 and one methyl group δ_C_ 33.5 in **1**, respectively, indicating that compound **2** was 3*β*,16*β*,22*α*,28-tetrahydroxyolean-11,13-dien-29-oic acid (**2a**) derivative. The molecular weight of **2** was 18 mass-units less than that of **2a**, suggesting that **2** was a dehydration derivative of 3*β*,16*β*,22*α*,28-tetrahydroxyolean-11,13-dien-29-oic acid. The closure of a γ-lactone ring between the hydroxy group at C-22 and the acyl carbon at C-29 was determined, based on the HMBC correlations (Figure S13) from H-22 (δ_H_ 5.10) to C-29 (δ_C_182.9). The NOESY correlations (Figure 3 and Figure S14) from H-22 to H-30 and H-19*β*, and from H-19*β* to H-30, confirmed the *α*-orientation of the γ-lactone ring at C-20 and C-22. From the above analysis, compound **2** was identified as 3*β*,16*β*,22*α*,28-tetrahydroxyolean-11,13-dien-22*α*,29-lactone, and named cissatriterpenoid B.

Compound **3** was obtained as a white amorphous powder. The IR spectrum displayed the presence of carbonyl (1770 cm^−1^) and hydroxy (3371 cm^−1^) groups. Its ^1^H and ^13^C NMR (Table 1, Figures S15 and S16) were quite analogous to those of **2**, with the obvious difference being the presence of an olefinic proton δ_H_ 5.44 (1H, brs), two olefinic carbons δ_C_ 126.7, 140.8 and two aliphatic olefinic carbons δ_C_ 24.6, 40.9 in **3** instead of four olefinic carbons δ_C_ 124.9, 129.9, 141.7, 126.8 in **2**. In the HMBC spectrum, the correlation (Figure 2 and Figure S20) of H-12 (δ_H_ 5.44) to C-11 (δ_C_ 24.6) and C-13 (δ_C_ 140.8), indicated that the olefinic bond was located at C-12 and C-13. This indicated that compound **3** was 3*β*,16*β*,22*α*,28-tetrahydroxyolean-12-ene-22*α*,29-lactone. This was further supported by their HR-ESI-MS, which gave a sodium adduct ion *m*/*z* 509.3242 (calcd 509.3243) in **3**, being 2 mass-units less than that of **2**. On the basis of the above evidence, compound **3** was identified as 3*β*,16*β*,22*α*,28-tetrahydroxyolean-12-ene-22*α*,29-lactone, and named cissatriterpenoid C.

By the MTS method, the cytotoxic activities of all isolated compounds were evaluated against HL-60, A549, SMMC-7721, MCF-7, and SW480 cell lines. Compound **3** showed the most potent cytotoxic activities against A549, SMMC-7721, MCF-7, and SW480 cell lines, with IC_50_ values of 17.55, 34.74, 19.77, and 30.39 μM, respectively, whereas the three remaining isolates were found to be inactive. By ChemBio 3D Ultra, the lipid-water partition coefficients of compounds **1**−**4** were determined as 7.57, 4.23, 4.65, and 6.45, respectively. Compounds **1**−**4** were tested against NO release effect in LPS-induced RAW 264.7 cells. Unfortunately, they showed no obvious inhibitory activity against NO production.

## 3. Experimental Section

### 3.1. General Experimental Procedures

Optical rotations were determined by a Rudolph AP-IV polarimeter (Rudolph, Hackettstown, NJ, USA). UV and IR spectra were obtained using a Thermo EVO 300 spectrometer (Thermo, Waltham, MA, USA) and a Thermo Nicolet IS 10 spectrometer (Thermo, Waltham, MA, USA), respectively. NMR and mass spectra were performed on a Bruker Avance III 500 spectrometer (Bruker, Rheinstetten, Germany) and a Bruker maXis HD mass spectrometer (Bruker, Bremen, Germany), respectively. Preparative HPLC separations were run on a SEP system (Beijing Sepuruisi Scientific Co., Ltd., Beijing, China) equipped with a variable-wavelength UV detector, using a YMC-Pack ODS-A column (250 × 20 mm, 5 μm). MCI gel CHP-20, sephadex LH-20 (40−70 μm), and silica gel (160−200 mesh) were acquired from TOSOH Corp., Tokyo, Japan, Amersham Pharmacia Biotech AB, Uppsala, Sweden, and Marine Chemical Industry, Qingdao, China, respectively. Chemical reagents for isolation were of analytical grade and purchased from Tianjin Siyou Co., Ltd., Tianjin, China. Biological reagents were from Sigma Company (St. Louis, MO, USA).

### 3.2. Plant Material

The whole plants of *C. pareira* var. *hirsuta* were collected in Yunnan province, China, in July 2018, and identified by Prof. Cheng-Ming Dong at School of Pharmacy, Henan University of Chinese Medicine, where a voucher specimen (SE 20180705) was deposited. Fresh whole plants were dried in the sun.

### 3.3. Extraction and Isolation

The dried whole plants of C. *pareira* var. *hirsuta* (50.7 kg) were powdered and refluxed with 95% EtOH (3 × 300 L) at 90 °C, and the combined solution was evaporated to dryness under reduced pressure to produce a crude extract (2.7 kg). The extract was dispersed in water (9 L) and successively partitioned with petroleum ether (PE, 9 L × 3), CH_2_Cl_2_ (9 L × 3), and n-BuOH (3.2 L × 3) to yield three extract fractions. The CH_2_Cl_2_ extract (740.1 g) was fractionated by silica gel column chromatography (CC, 125 × 15 cm) with PE (60–90 °C)–acetone (100:0–1:2) to obtain five fractions (A1–A5) based on TLC analysis. Fraction A4 (10.32 g) was subjected to MCI gel CHP-20 CC (23 × 4 cm) eluted with methanol–H_2_O (10:90–80:20) to generate five subfractions (A4–1~A4–5). Subfraction A4–3 (2.17 g) was performed with silica gel CC (45 × 5 cm) eluted by PE–EtOAC (10:1–1:2) to afford five subfractions (A4–3–1~A4–3–5). Subfraction A4-3-3 (936.2 mg) was further purified by preparative HPLC (CH_3_CN–H_2_O, 50:50) at a flow rate of 6 mL min^–1^ to produce **2** (3.4 mg, tR 80.1 min, 98.7%) and **3** (9.5 mg, tR 57.0 min, 99.0%). Subfraction A4–4 (1.36 g) was separated in silica gel CC (45 × 5 cm) eluted by PE–EtOAC (10:1–1:2) to give four subfractions (A4–4–1~A4–4–4). Subfraction A4–4–4 (686.6 mg) was rechromatographed by sephadex LH-20 CC (100 × 2.5 cm) eluted by methanol to provide three subfractions (A4–4–4–1~A4–4–4–3). Sub-fraction A4–4–4–3 was subjected to preparative HPLC eluted with CH_3_CN–H_2_O (60:40) at a flow rate of 6 mL min^–1^ to give **4** (20.0 mg, tR 42.2 min, 99.1%). Subfraction A4–5 (3.32 g) was submitted to silica gel CC (45 × 5 cm) eluted by PE–EtOAC (10:1–1:2) to obtain three subfractions (A4–5–1~A4–5–3). Further separation of subfraction A4–5–2 (988.5 mg) using Sephadex LH-20 CC (MeOH) resulted in five subfractions A4–5–2–1~A4–5–2–5. Subfraction A4–5–2–3 (349.8 mg) was purified by preparative HPLC eluted with MeOH–H_2_O (75:25) at a flow rate of 6 mL min^–1^ to give **1** (20.1 mg, tR 96.3 min, 98.4%).

### 3.4. Spectroscopic and Physical Data

Cissatriterpenoid A (**1**): white, amorphous powder; [α${]}_{D}^{20}$ − 7.0 (c 0.03, MeOH); UV (MeOH) λmax (log ε) 218 (4.09), 264 (3.64) nm; IR (iTR)ν_max_ 3341, 2942, 1726, 1593, 1461, 1384, 1329, 1284, 1114, 1027 cm^−1^; HR-ESI-MS (positive): *m*/*z* 618.3559 [M + K]^+^ (calcd for C_36_H_53_NO_5_K, 618.3561); NMR data (CD_3_OD), see Table 1.

Cissatriterpenoid B (**2**): white, amorphous powder; [α${]}_{D}^{20}$ + 18.2 (c 0.07, MeOH); UV (MeOH) λmax (log ε) 206 (3.53) nm; IR (iTR)ν_max_ 3383, 2932, 1766, 1454, 1384, 1274, 1182, 1074, 1035 cm^−1^; HR-ESI-MS (positive): *m*/*z* 507.3079 [M + Na]^+^ (calcd for C_30_H_44_O_5_Na, 507.3086); NMR data (CD_3_OD), see Table 1.

Cissatriterpenoid C (**3**): white, amorphous powder; [α${]}_{D}^{20}$ + 11.6 (c 0.01, MeOH); UV (MeOH) λmax (log ε) 201 (3.75) nm; IR (iTR)ν_max_ 3371, 2936, 1770, 1453, 1381, 1185, 1077, 1038 cm^−1^; HR-ESI-MS (positive): *m*/*z* 509.3242 [M + Na]^+^ (calcd for C_30_H_46_O_5_Na, 509.3243); NMR data (CD_3_OD), see Table 1.

### 3.5. Microhydrolysis of Compound **1**

Compound **1** (10 mg) was dissolved in 1.0 mL of MeOH, then 0.3 mL of 28% sodium methylate solution was added dropwise under an N_2_ atmosphere. After 4 h stirring at room temperature, the reaction mixture was neutralized by dilute HCl, and extracted with CH_2_Cl_2_. The extract was concentrated under reduced pressure, and isolated by preparative HPLC eluted with CH_3_CN-H_2_O (60:40) at a flow rate of 6 mL min^–1^ to obtain chichipegenin (tR 42.2 min) [12].

### 3.6. Cytotoxicity Asssay

Using the MTS method previously described [15], the cytotoxic activities of compounds 1–4 were evaluated against human myeloid leukemia HL-60, lung cancer A-549, hepatocellular carcinoma SMMC-7721, breast cancer MCF-7, and colon cancer SW-480 cell lines. All cells were cultured in RPMI-1640 medium, supplemented with 10% fetal bovine serum (FBS) at 37 °C under 5% CO_2_ in a humidified atmosphere. Cell viability was assessed by conducting colorimetric measurements of the amount of insoluble formazan formed in living cells based on the reduction of 3-(4,5-dimethylthiazol-2-yl)-5-(3-carboxymethoxyphenyl)-2-(4-sulfophenyl)-2H-tetrazolium (MTS). In brief, 100 µL of adherent cells was seeded into each well in a 96-well cell culture plate and kept for 24 h for adherence. Each tumor cell line was exposed to various concentrations of the test compound in triplicate for 48 h. After incubation, MTS (20 μL) was added to each well, and the incubation continued for 4 h at 37 °C. The absorbance was measured at 492 nm in a 96-well microtiter plate reader. The IC_50_ value of each compound was calculated by the Reed-Muench’s method. Cisplatin was used as positive control.

### 3.7. NO Inhibitory Activity

NO production in the murine monocytic RAW 264.7 macrophage was evaluated by the previously reported protocol [16]. L-NG-Monomethyl arginine (LNMMA) was used as a positive control. The RAW 264.7 macrophages were cultured in DMEM medium containing 10% FBS and penicillin-streptomycin (100 U/mL) at 37 °C under 5% CO_2_. The test compounds were dissolved in DMSO and then diluted to different concentrations by medium. Cells were precultured in 96-well plates (2 × 10^5^ cells/well) for 24 h, and then incubated with serial dilutions of the test compounds and LPS (1.0 μg/mL) for 18 h. Then, 100 μL of Griess reagent and 100 μL of culture supernatant were mixed, and incubated for 5 min. The optical density of the mixture was read at 570 nm by an automated microplate reader. The NO inhibitory rate was determined via a comparison with the control group.

## 4. Conclusions

Phytochemical investigation of *C. pareira* var. *hirsuta* resulted in the isolation of three new polyhydroxylated oleanane triterpenoids, cissatriterpenoid A−C (**1**−**3**), and one known analogue (**4**). To date, more than 500 polyhydroxylated oleanane triterpenoids have been found in the families Cyrillaceae, Hippocastanceae, Loganiaceae, Myrsinaceae, Pittosporaceae, Sapindaceae, Symplocaeae, Styracaceae, Theaceae, Lecythidaceae, and Umbelliferae since 1934 [17]. Compounds **1**–**4** represent the first report of polyhydroxylated oleanane triterpenoids from the family Menispermaceae. The hydroxyl groups in the structure skeleton provide numerous acylation and glycosylation bonding sites, and facilitate interactions with molecular targets [17]. This also lays a chemical-substance foundation for pharmacological research of *C. pareira* var. *hirsuta*. The cytotoxic activities of all isolated compounds were tested against HL-60, A549, SMMC-7721, MCF-7, and SW480 cell lines. Compounds **2**–**4** have the same structural skeleton, so the variation in cytotoxicity between them indicates that the γ-lactone ring at C-22 and C-29, and the olefinic bond at C-12 and C-13 are structurally required for the cytotoxicity of polyhydroxylated oleanane triterpenoids against the A549, SMMC-7721, MCF-7, and SW480 cell lines. Based on the lipid-water partition coefficients, compounds **1**–**4** were relatively hydrophobic. Furthermore, compound **3** is less lipophilic than **1** and **4**, which agrees with their cytotoxic activities. These results reveal that compound **3** may be a potential anticancer lead. Further studies are necessary to explore the anticancer mechanism and cytotoxicity in normal cells. Polyhydroxylated oleanane triterpenoids have been reported to modulate specific signaling pathways and inhibit cancer cell proliferation [17], including HT-29, HCT-116, HepG2, Bel-7402, SMMC-7721, SNU-432, A2780/CP70, OVCAR-3, HSC-20, C6, U251, U87-MG, A549, QBC939, Sk-ChA-1, MZ-ChA-1, HL-60, A375.S2, 786-O, Caki-1, PC-3, DU-145, AGS, SNU-719, and HCC-1428 cell lines. Therefore, they will become the main anticancer effective components in the genus *Cissampelos*. This study not only extends the chemical-structure diversity of oleanane triterpenoids, but also provides a direction for identifying potential anticancer secondary metabolites from traditional Chinese medicine.

## Figures and Tables

**Table 1:** ^1^H NMR and ^13^C NMR Data (500 MHz, CD_3_OD) of **1**−**3**.

NO.	1		2		3	
	δ_H_	δ_C_	δ_H_	δ_C_	δ_H_	δ_C_
1	1.63, m; 1.00, m	39.9 CH_2_	1.90, m; 1.05, m	39.2 CH_2_	1.66, m; 1.01, m	39.9 CH_2_
2	1.64, m; 1.56, m	27.8 CH_2_	1.67, m; 1.64, m	27.7 CH_2_	1.65, m; 1.58, m	27.9 CH_2_
3	3.14, dd (11.3, 4.7)	79.6 CH	3.17, dd (11.5, 5.0)	79.6 CH	3.16, dd (11.2, 4.1)	79.6 CH
4		39.9 C		40.0 C		39.9 C
5	0.77, d (12.8)	56.6 CH	0.84, d (11.9)	56.2 CH	0.79, d (11.4)	56.7 CH
6	1.62, m; 1.49, m	19.4 CH_2_	1.67, m; 1.50, m	19.4 CH_2_	1.60, m; 1.46, m	19.4 CH_2_
7	1.62, m; 1.39, m	33.8 CH_2_	1.42, m; 0.98, m	33.4 CH_2_	1.57, m; 1.43, m	34.3 CH_2_
8		41.2 C		41.2 C		40.6 C
9	1.57, dd (11.9, 7.2)	48.2 CH	1.92, m	55.3 CH	1.54, m	48.1 CH
10		38.0 C		37.7 C		38.1 C
11	1.89, m	24.7 CH_2_	6.32, dd (10.7, 2.9)	124.9 CH	1.92, m	24.6 CH_2_
12	5.27, t (3.4)	125.5 CH	5.72, d (10.7)	129.9 CH	5.44, brs	126.7 CH
13		142.3 C		141.7 C		140.8 C
14		43.5 C		45.2 C		45.1 C
15	1.77, m; 1.38, m	36.3 CH_2_	1.86, m; 1.42, m	34.5 CH_2_	1.90, m; 1.35, m	35.3 CH_2_
16	4.77, dd (11.7, 5.4)	67.5 CH	4.19, dd (12.4, 4.8)	69.9 CH	4.21, dd (11.8, 3.6)	67.8 CH
17		44.9 C		49.6 C		44.2 C
18	2.62, dd (14.0, 4.7)	44.7 CH		126.8 C	2.60, dd (11.4, 10.0)	40.9 CH
19	1.87, m	46.8 CH_2_	2.50, m; 2.25, m	39.1 CH_2_	1.85, m; 1.61, m	40.5 CH_2_
20		32.8 C		44.1 C		40.6 C
21	1.72, m; 1.63, m	44.1CH_2_	2.69, dd (14.9, 1.9); 2.26, dd (14.9)	35.9 CH_2_	2.45, d (12.2); 2.05, dd (11.9, 5.1)	35.7 CH_2_
22	4.32, dd (12.6, 4.7)	70.9 CH	5.10, d (6.0)	79.0 CH	4.90, d (5.3)	80.1 CH
23	0.98, s	28.7 CH_3_	0.98, s	28.4 CH_3_	0.98, s	28.7 CH_3_
24	0.78, s	16.3 CH_3_	0.77, s	15.8 CH_3_	0.79, s	16.4 CH_3_
25	0.96, s	16.1 CH_3_	0.91, s	18.6 CH_3_	0.98, s	16.2 CH_3_
26	1.08, s	17.5 CH_3_	0.75, s	17.2 CH_3_	0.98, s	17.5 CH_3_
27	1.30, s	27.9 CH_3_	0.98, s	22.6 CH_3_	1.17, s	25.6 CH_3_
28	4.68, d (11.0); 4.55, d (11.0)	64.1 CH_2_	3.95, d (12.1); 3.74, d (12.1)	65.0 CH_2_	3.85, d (11.1); 3.40, d (11.1)	64.2 CH_2_
29	0.98, s	33.5 CH_3_		182.9 C		185.2 C
30	1.03, s	25.1 CH_3_	1.22, s	20.4 CH_3_	1.17, s	21.2 CH_3_
1′		166.2 C				
3′	9.14, brs	150.9 CH				
4′		128.1 C				
5′	8.40, dt (8.0, 1.9)	138.8 CH				
6′	7.59, dd (8.0, 5.1)	125.4 CH				
7′	8.76, d (3.9)	154.2 CH				

## Data Availability

Data are contained within the manuscript.

## Supplementary Materials

Figures S1–S21: 1H-NMR, ^13^C-NMR, DEPT, ^1^H-^1^H COSY, HSQC, HMBC, and NOESY spectra of compounds **1**–**3**.

## Sample Availability

Isolated compounds are not available from the authors.
