# Supplementary material for: Cytotoxic Polyhydroxylated Oleanane Triterpenoids from Cissampelos pareira var. hirsuta
Source: Molecules. 2022 Feb 10;27(4):1183. doi: 10.3390/molecules27041183 (PMC8876210; doi:10.3390/molecules27041183)
Supplement: Supplementary file 1 [file molecules-27-01183-s001.zip › molecules-1572209-supplementary.pdf]

Cytotoxic Polyhydroxylated Oleanane Triterpenoids from *Cissampelos pareira* var. *hirsuta*

Yanjun Sun [1,2,3*], Ruyi Pan [1,2], Haojie Chen [1,2], Chen Zhao [1,2], Ruijie Han [1,2], Meng Li [1,2], Guimin Xue [1,2], Hui Chen [1,2], Kun Du [1,2], Junmin Wang [1,2], and Weisheng Feng [1,2*]

[1] Collaborative Innovation Center for Respiratory Disease Diagnosis and Treatment & Chinese Medicine, Development of Henan Province, Henan University of Chinese Medicine, Zhengzhou 450046, China
[2] School of Pharmacy, Henan University of Chinese Medicine, Zhengzhou 450046, China
[3] Henan Research Center for Special Processing Technology of Chinese Medicine, 450046, China

* Correspondence: sunyanjun2011@hactcm.edu.cn (Y.S.); fwsh@hactcm.edu.cn (W.F.);
Tel.: +86-371-6596-2746 (Y.S. & W.F.)

Abstract: Three new polyhydroxylated oleanane triterpenoids, cissatriterpenoid A−C (**1**–**3**), along with one known analogue (**4**), were isolated from the whole plant of *Cissampelos pareira* var. *hirsuta*. Their chemical structures were elucidated by extensive spectroscopic data (IR, HR-ESI-MS, ^{1}H-NMR, ^{13}C-NMR, DEPT, ^{1}H-^{1}H COSY, HSQC, HMBC, NOESY) and microhydrolysis method. The isolation of compounds **1–4** represents the first report of polyhydroxylated oleanane triterpenoids from the family Menispermaceae. All isolated compounds were evaluated for their cytotoxicity against five human cancer cell lines, and inhibitory activity against NO release in LPS-induced RAW 264.7 cells. Compound **3** showed the most potent cytotoxic activities against the A549, SMMC-7721, MCF-7, and SW480 cell lines, with IC_{50} values of 17.55, 34.74, 19.77, and 30.39 μM, respectively, whereas three remaining ones were found to be inactive. The preliminary structure-activity relationship analysis indicated the γ-lactone ring at C-22 and C-29, and the olefinic bond at C-12 and C-13 were structurally required for the cytotoxity of polyhydroxylated oleanane triterpenoids against these four cell lines. Based on lipid-water partition coefficients, compound **3** is less lipophilic than **1** and **4**, which agrees with their cytotoxic activities. This confirms the potential of *C. pareira* var. *hirsuta* in the tumor treatment.

Keywords: *Cissampelos pareira* var. *hirsuta*; polyhydroxylated triterpenoids; oleanane; cytotoxic

Table of Contents

Figure S1. ^{1}H NMR spectrum (500 MHz) of **1** in CD_3OD 3
Figure S2. ^{13}C NMR spectrum (125 MHz) of **1** in CD_3OD 3
Figure S3. DEPT spectrum of **1** in CD_3OD 4
Figure S4. ^{1}H-^{1}H-COSY spectrum of **1** in CD_3OD 4
Figure S5. HSQC spectrum of **1** in CD_3OD 5
Figure S6. HMBC spectrum of **1** in CD_3OD 5
Figure S7. NOESY spectrum of **1** in CD_3OD 6
Figure S8. ^{1}H NMR spectrum (500 MHz) of **2** in CD_3OD 6
Figure S9. ^{13}C NMR spectrum (125 MHz) of **2** in CD_3OD 7
Figure S10. DEPT spectrumof **2** in CD_3OD 7
Figure S11. ^{1}H-^{1}H-COSY (500 MHz) spectrum of **2** in CD_3OD 8
Figure S12. HSQC (500 MHz) spectrum of **2** in CD_3OD 8
Figure S13. HMBC (500 MHz) spectrum of **2** in CD_3OD 9
Figure S14. NOESY (500 MHz) spectrum of **2** in CD_3OD 9
Figure S15. ^{1}H NMR spectrum (500 MHz) of **3** in CD_3OD 10
Figure S16. ^{13}C NMR spectrum (125 MHz) of **3** in CD_3OD 10
Figure S17. DEPT spectrum of **3** in CD_3OD 11
Figure S18. ^{1}H-^{1}H-COSY spectrum of **3** in CD_3OD 11
Figure S19. HSQC spectrum of **3** in CD_3OD 12
Figure S20. HMBC spectrum of **3** in CD_3OD 12
Figure S21. NOESY spectrum of **3** in CD_3OD 13

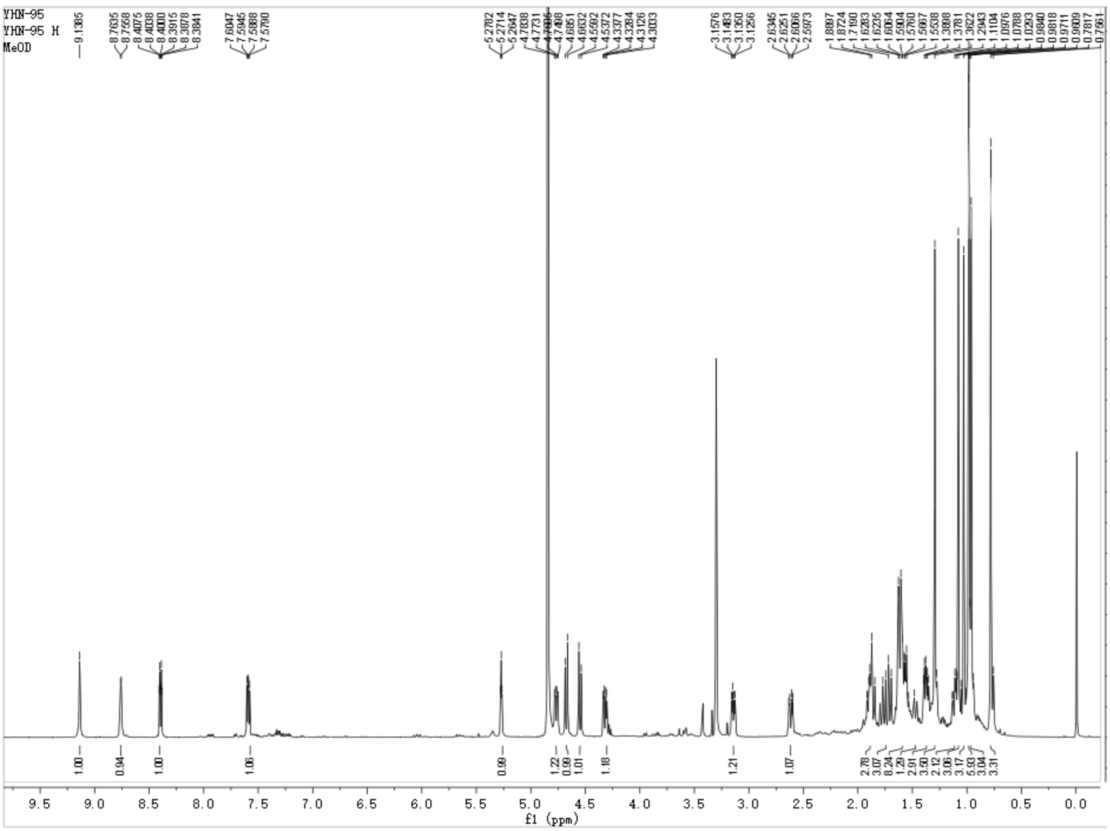

Figure S1. ^{1}H NMR spectrum (500 MHz) of **1** in CD_3OD

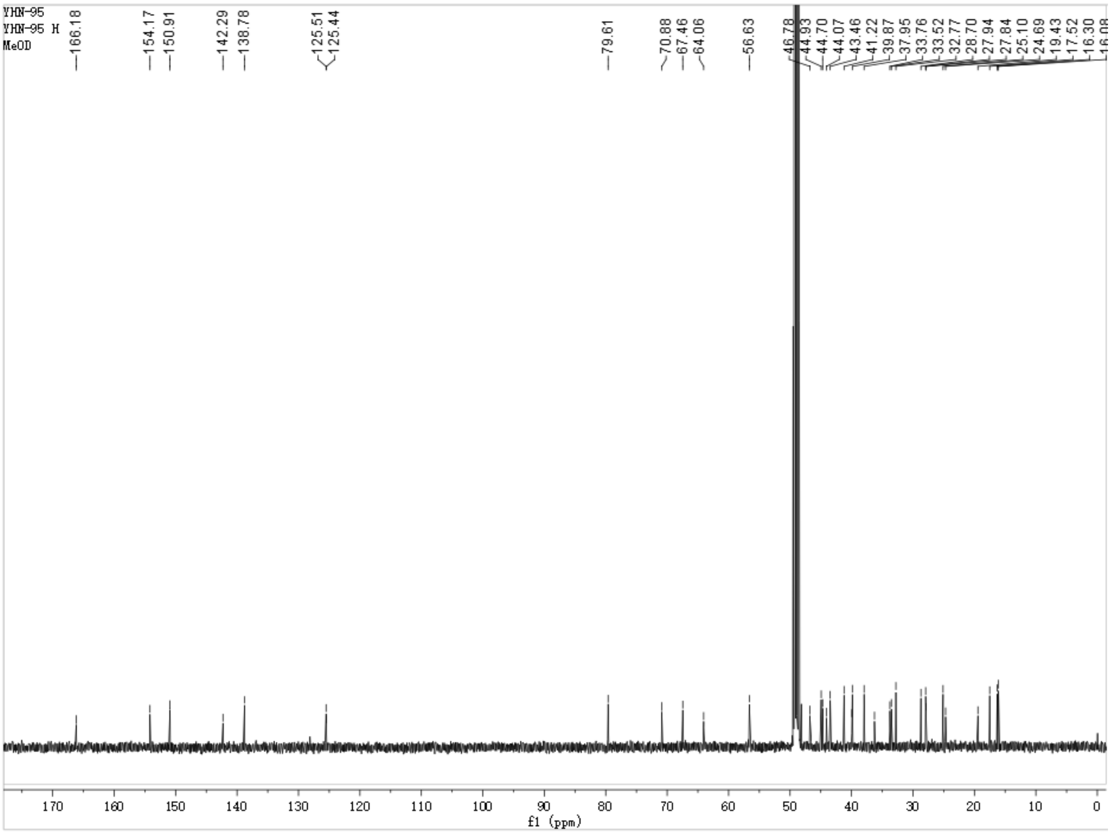

Figure S2. ^{13}C NMR spectrum (125 MHz) of **1** in CD_3OD

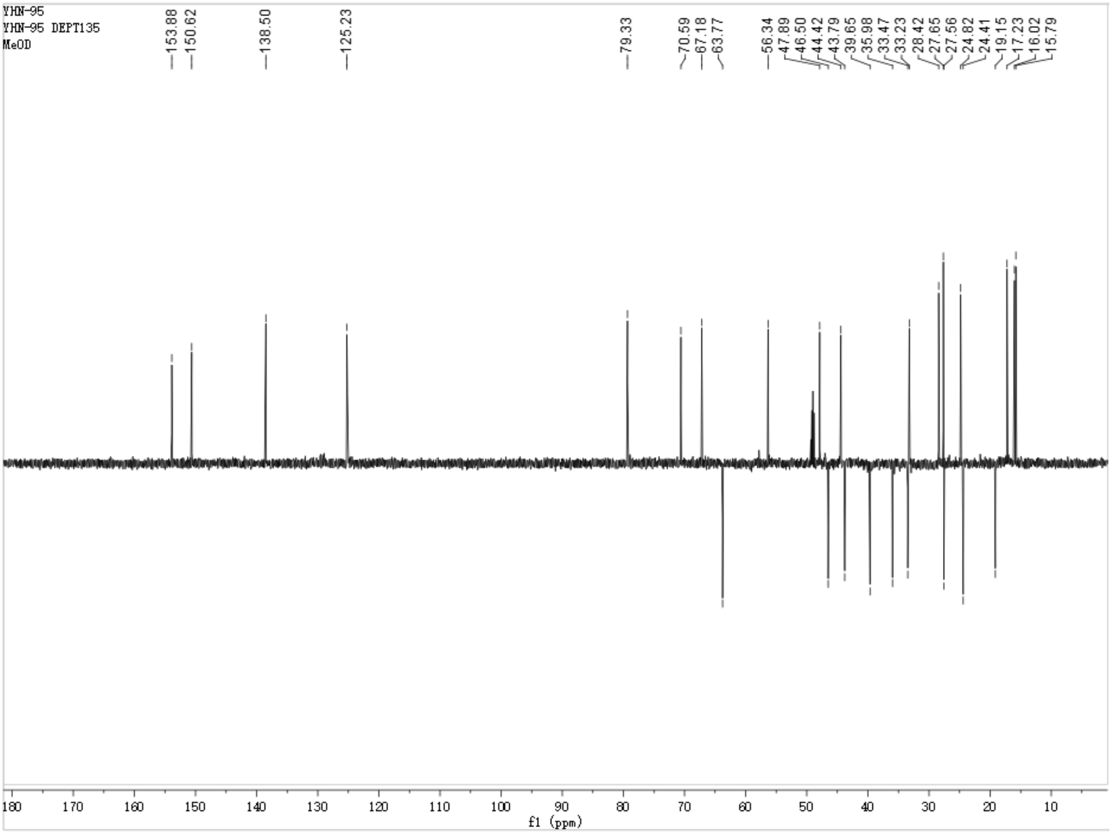

Figure S3. DEPT spectrum of **1** in CD_3OD

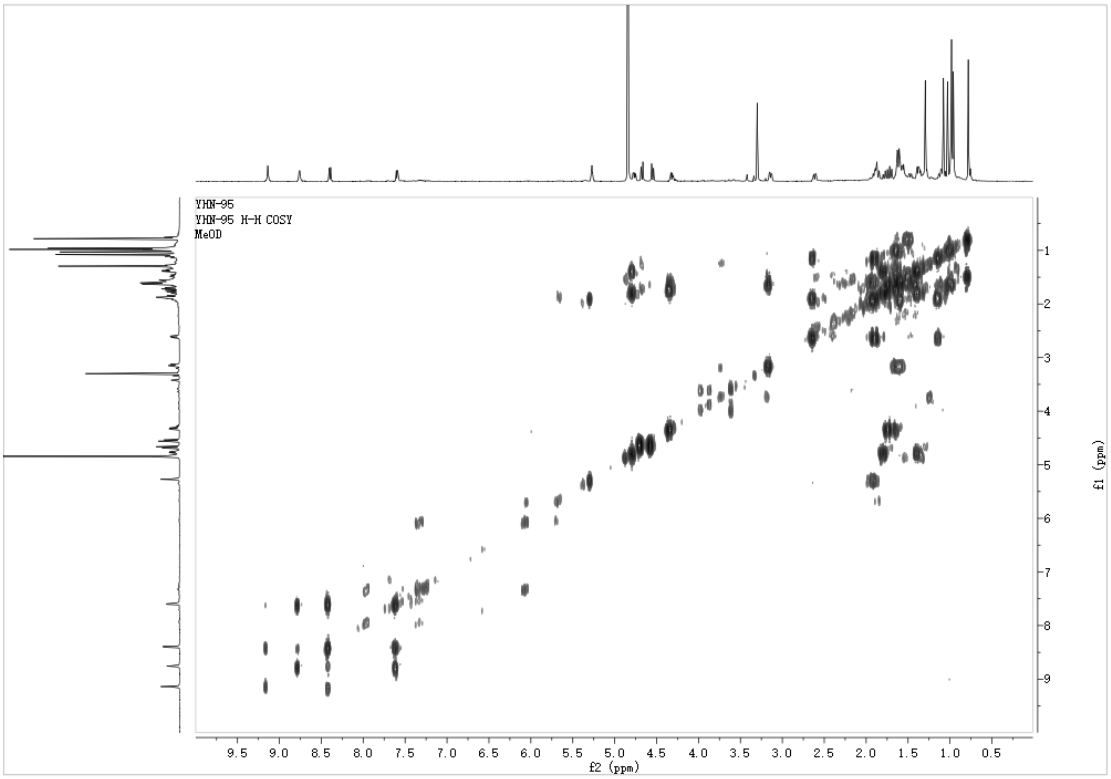

Figure S4. ^1H-^1H COSY spectrum of **1** in CD_3OD

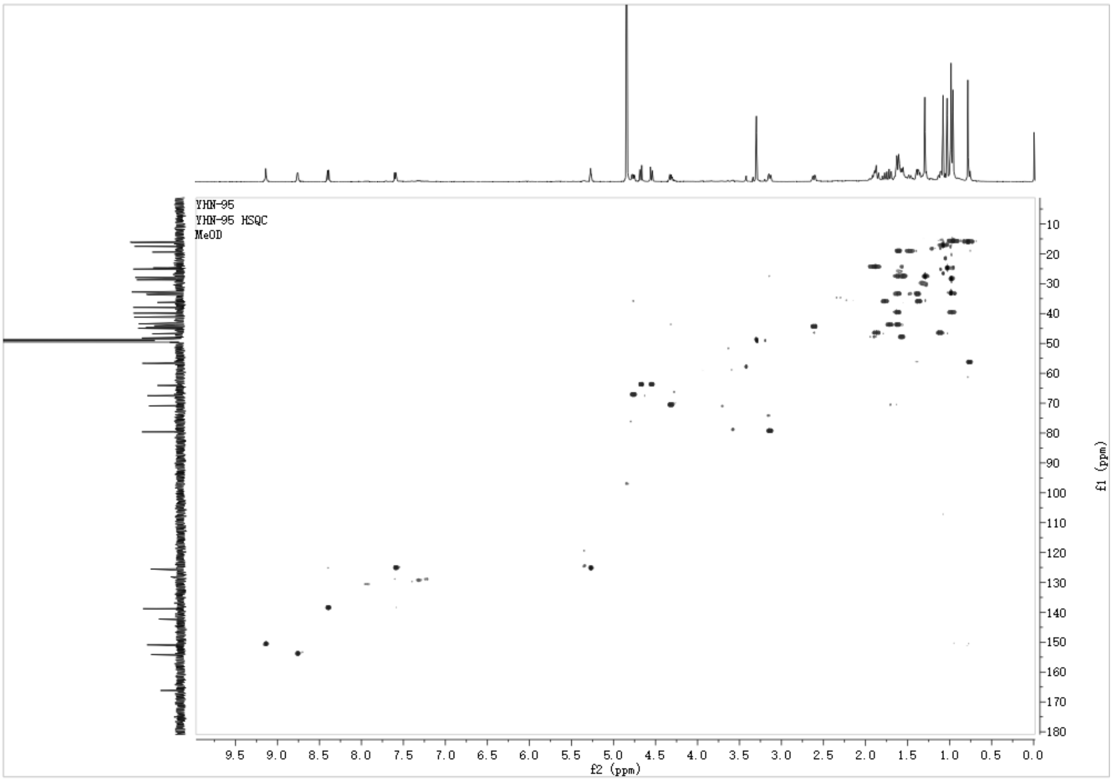

Figure S5. HSQC spectrum of **1** in CD_3OD

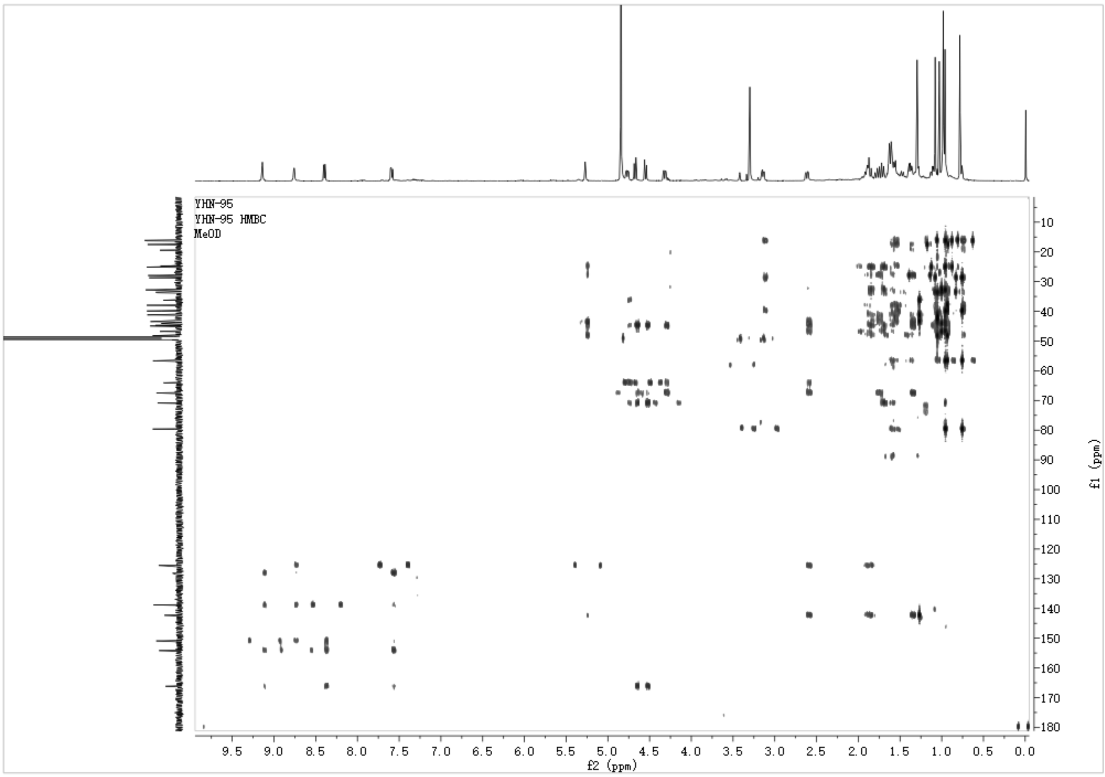

Figure S6. HMBC spectrum of **1** in CD_3OD

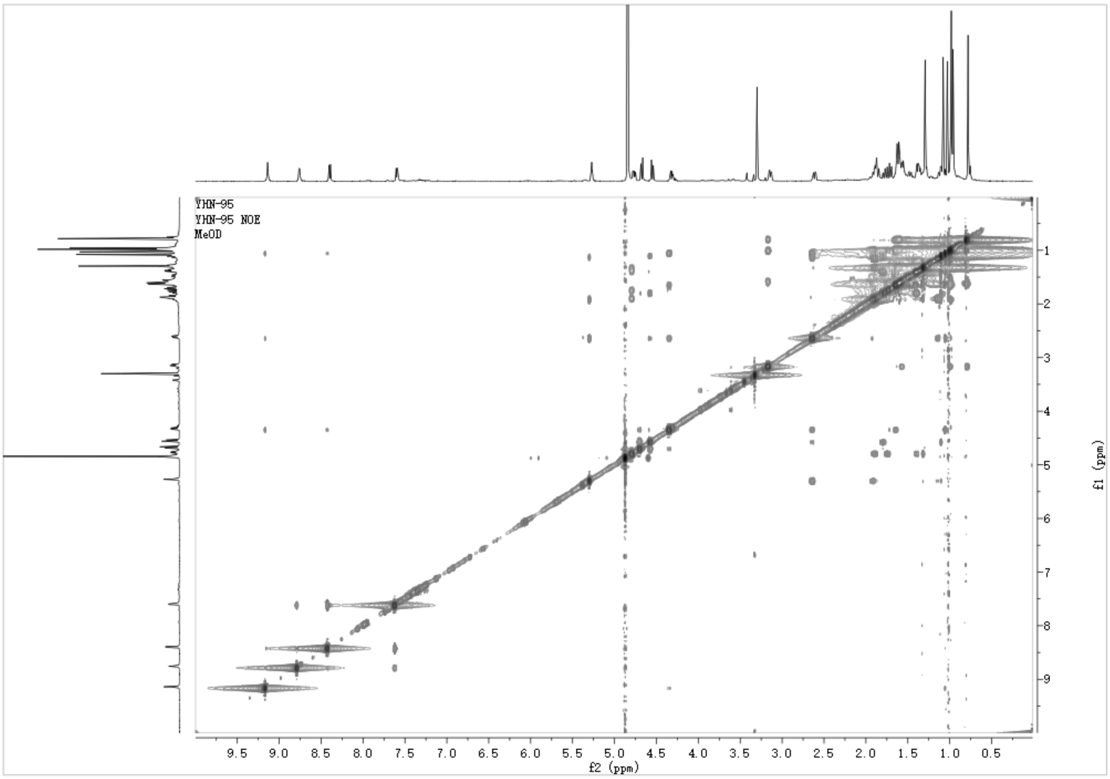

Figure S7. NOESY spectrum of **1** in CD_3OD

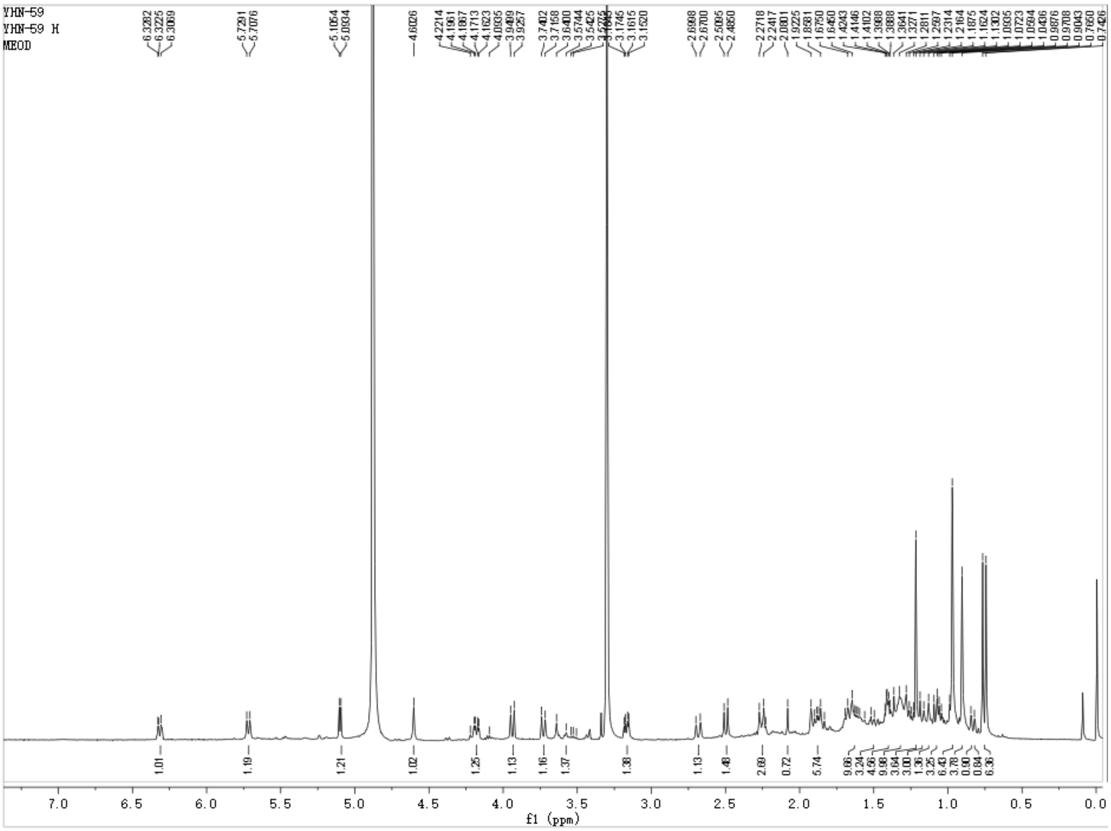

Figure S8. ^{1}H NMR spectrum (500 MHz) of **2** in CD_3OD

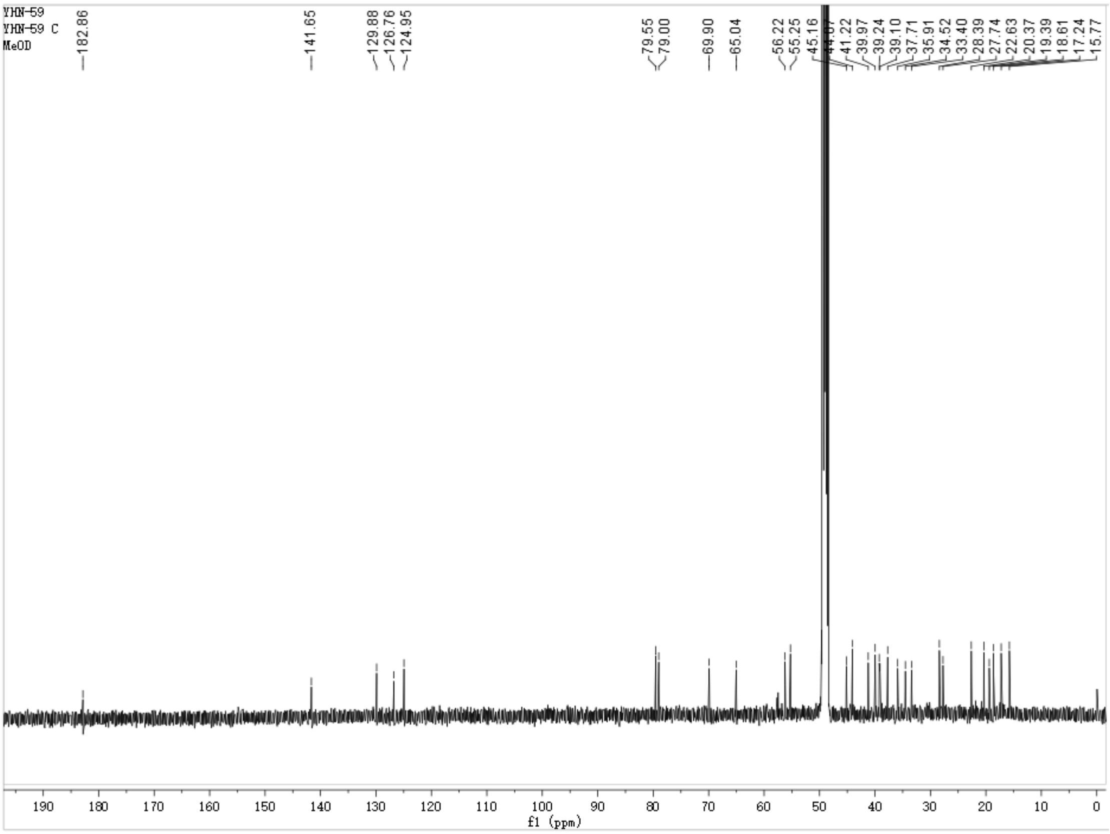

Figure S9. ^{13}C NMR spectrum (125 MHz) of **2** in CD_3OD

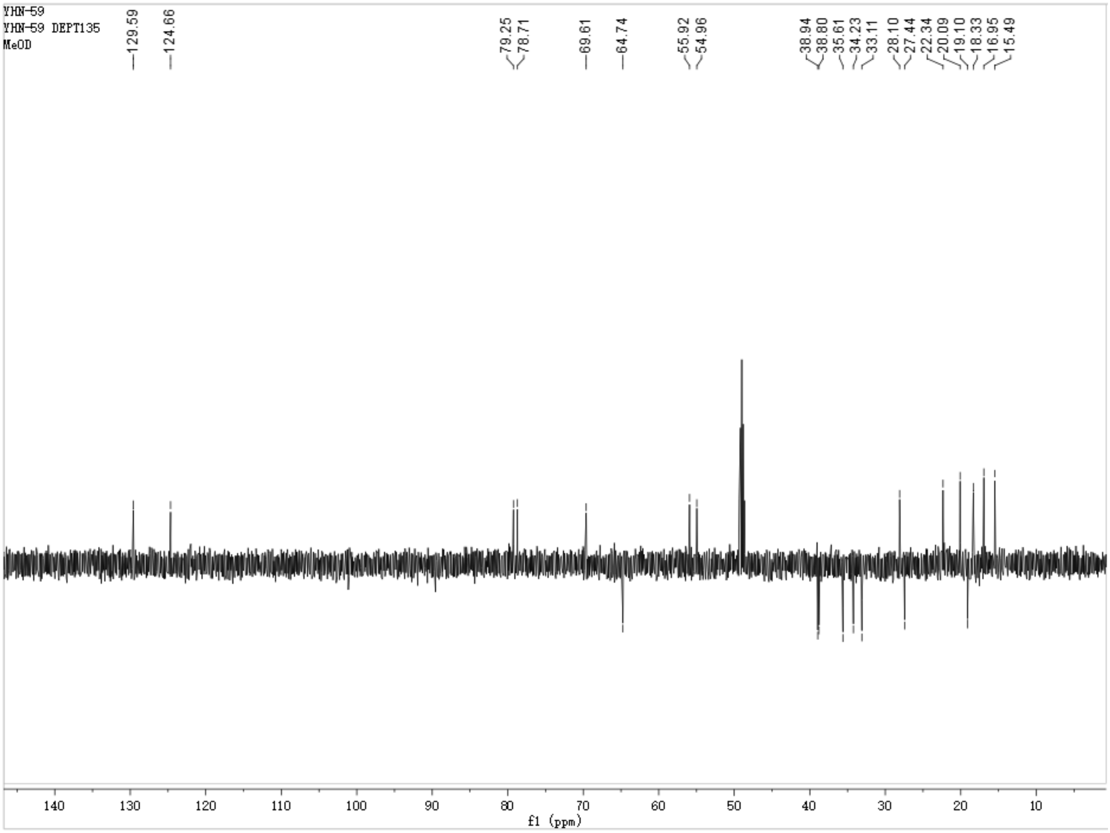

Figure S10. DEPT spectrum of **2** in CD_3OD

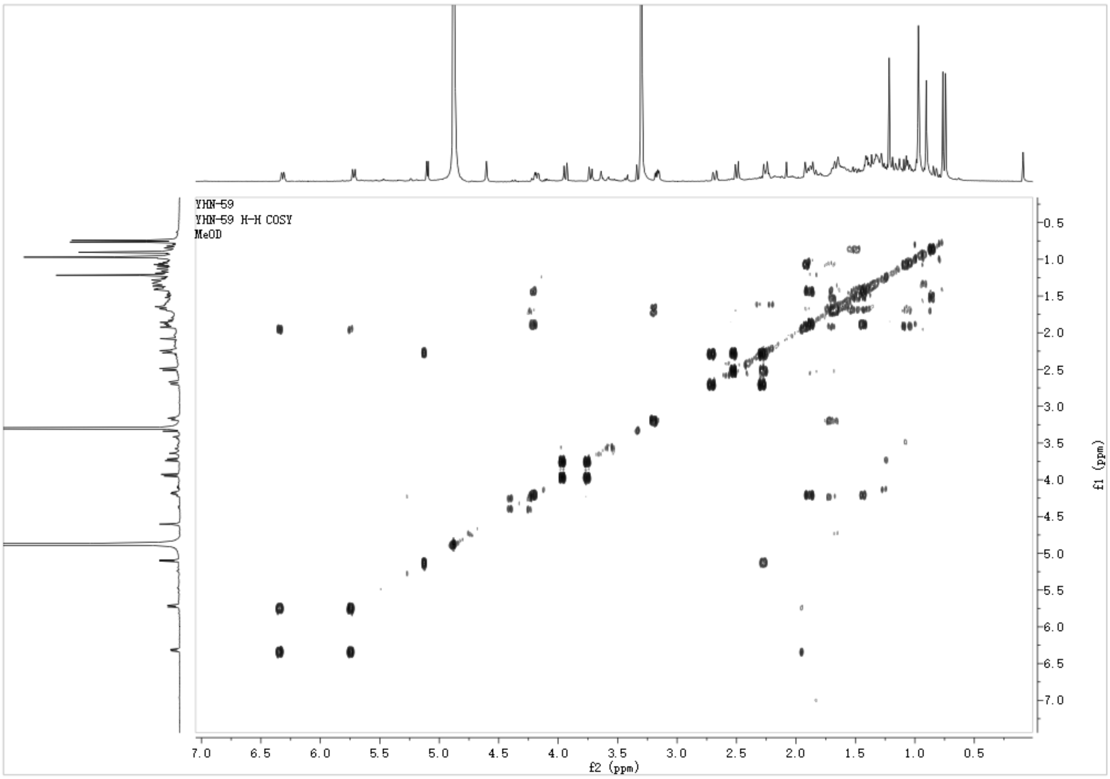

Figure S11. ^{1}H-^{1}H COSY spectrum of **2** in CD_3OD

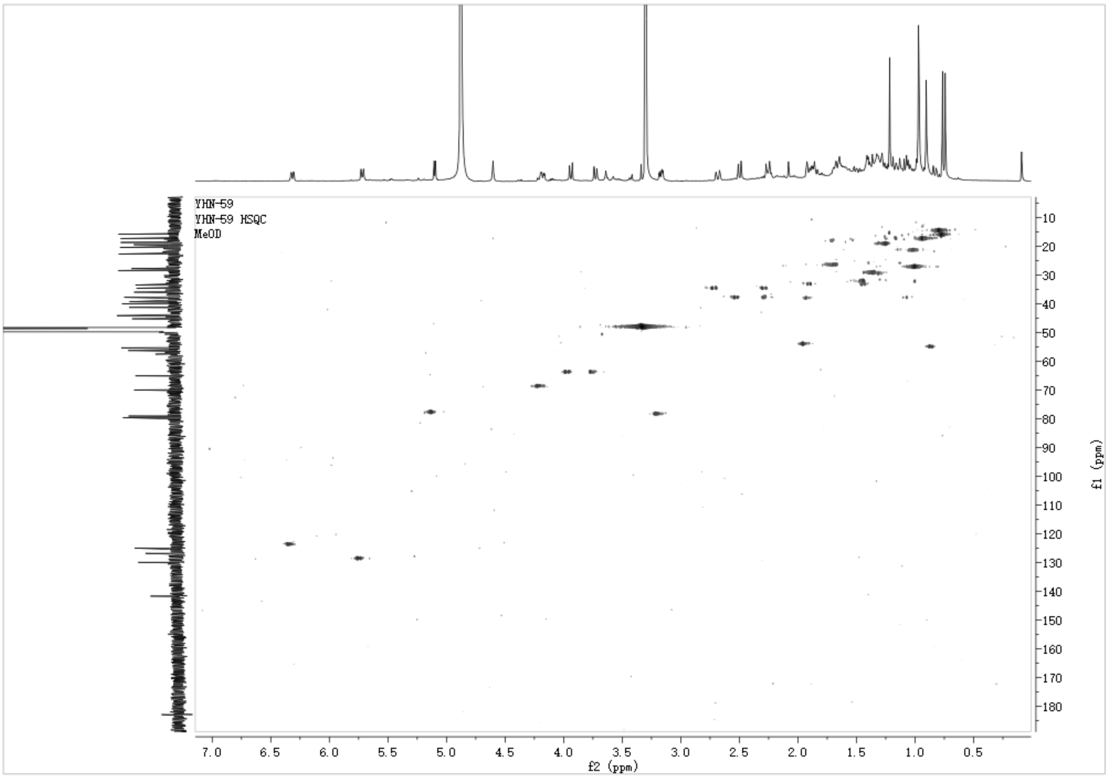

Figure S12. HSQC spectrum of **2** in CD_3OD

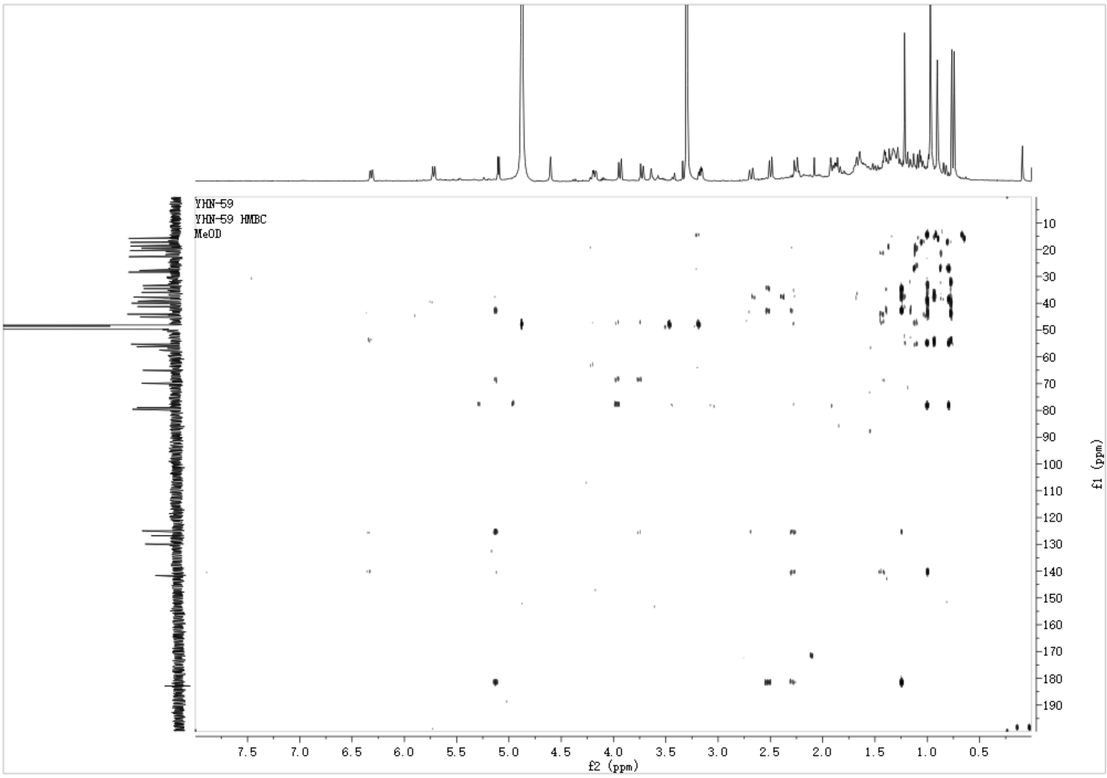

Figure S13. HMBC spectrum of **2** in CD_3OD

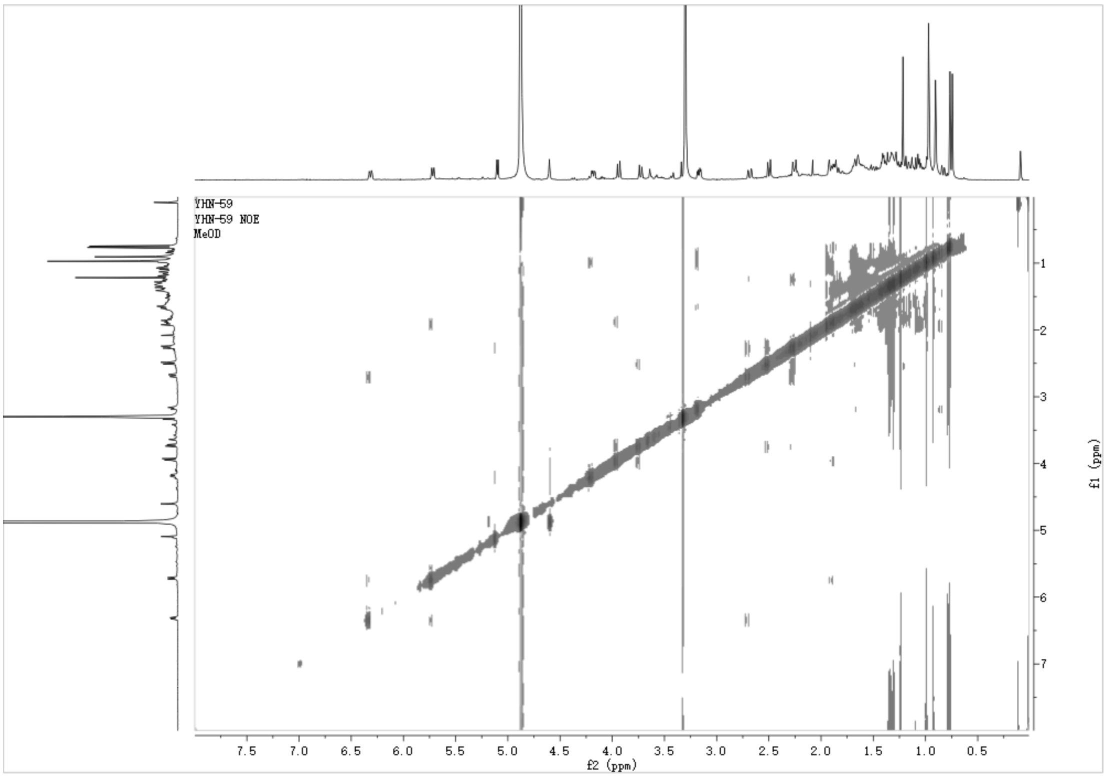

Figure S14. NOESY spectrum of **2** in CD_3OD

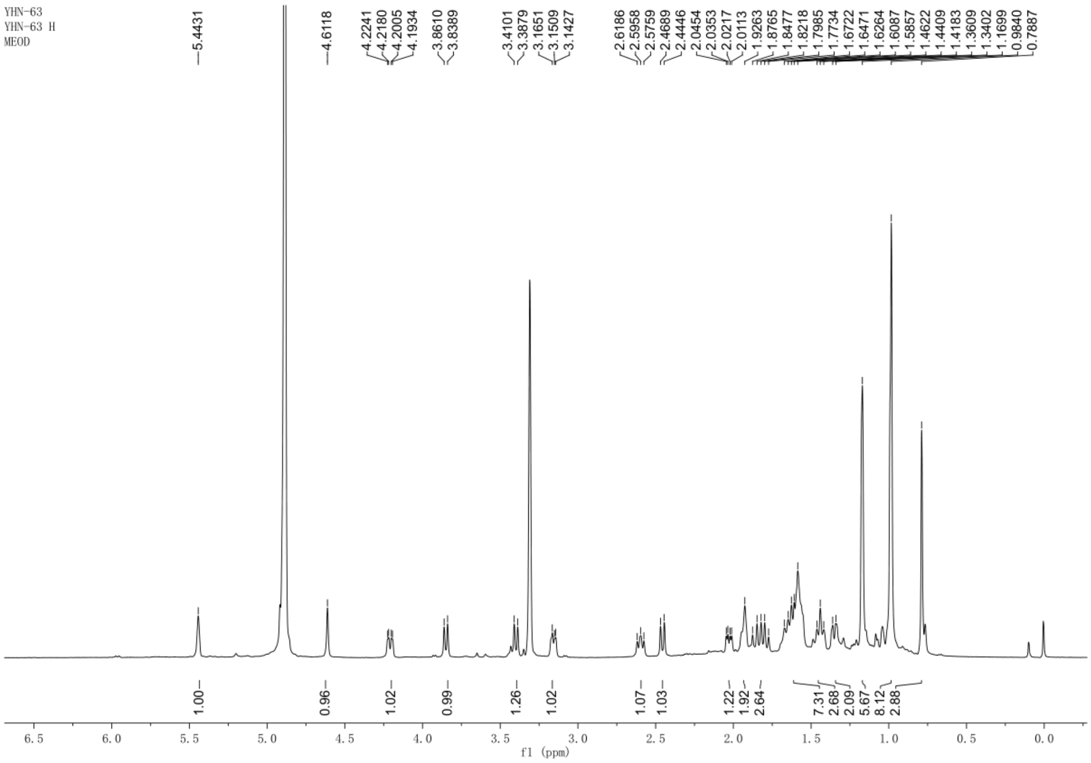

Figure S15. ^{1}H NMR spectrum (500 MHz) of **3** in CD_3OD

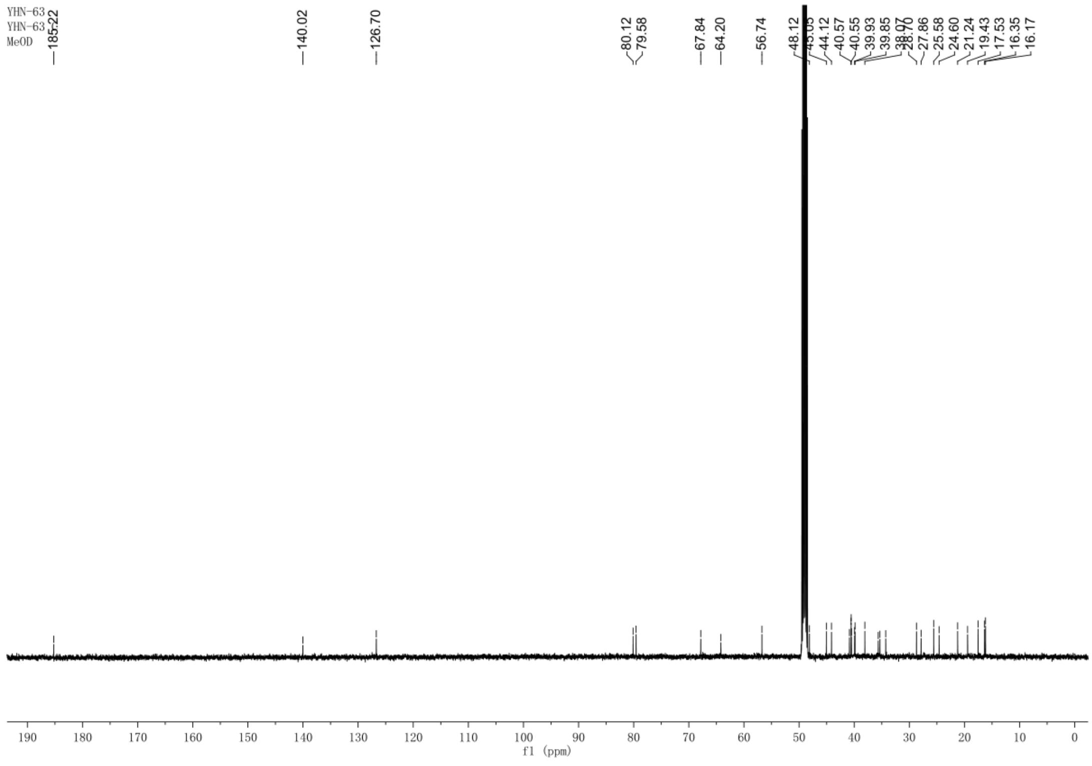

Figure S16. ^{13}C NMR spectrum (125 MHz) of **3** in CD_3OD

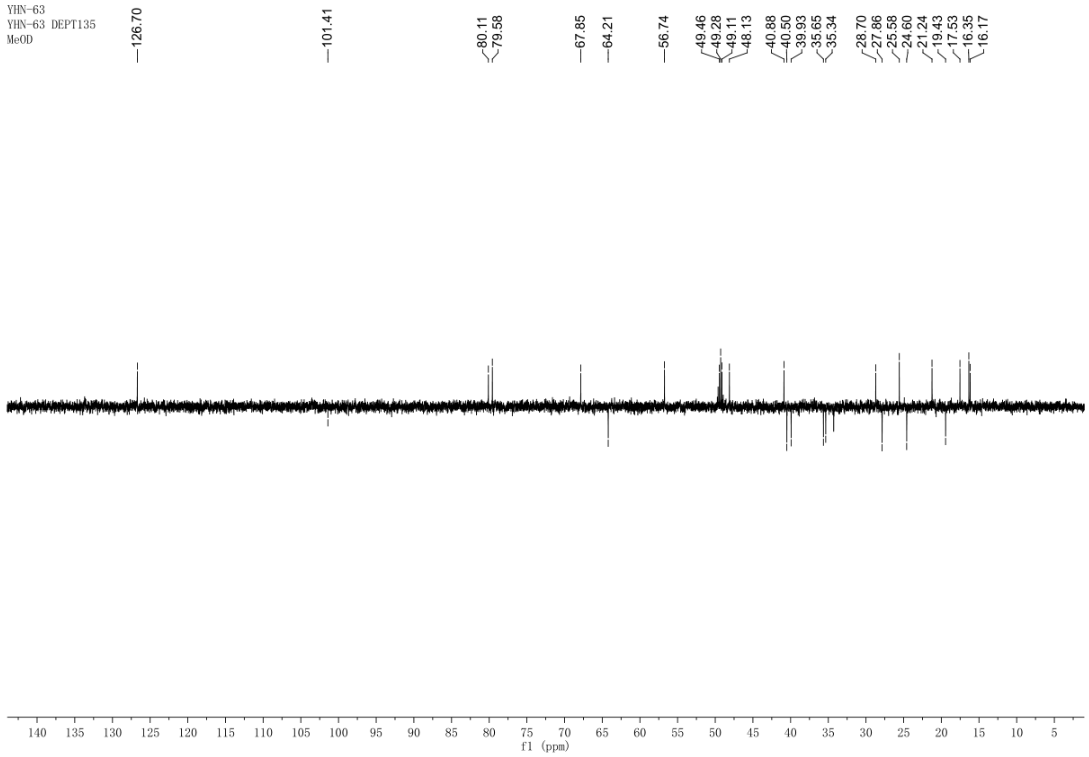

Figure S17. DEPT spectrum of **3** in CD_3OD

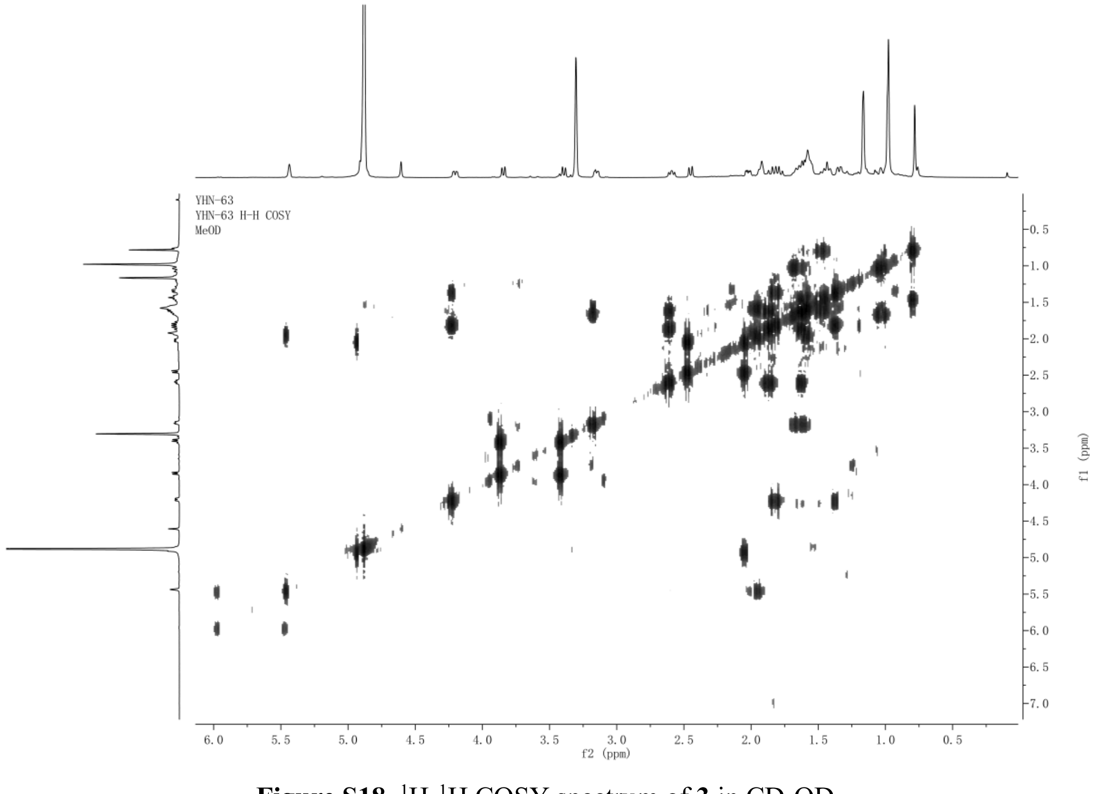

Figure S18. ^1H-^1H COSY spectrum of **3** in CD_3OD

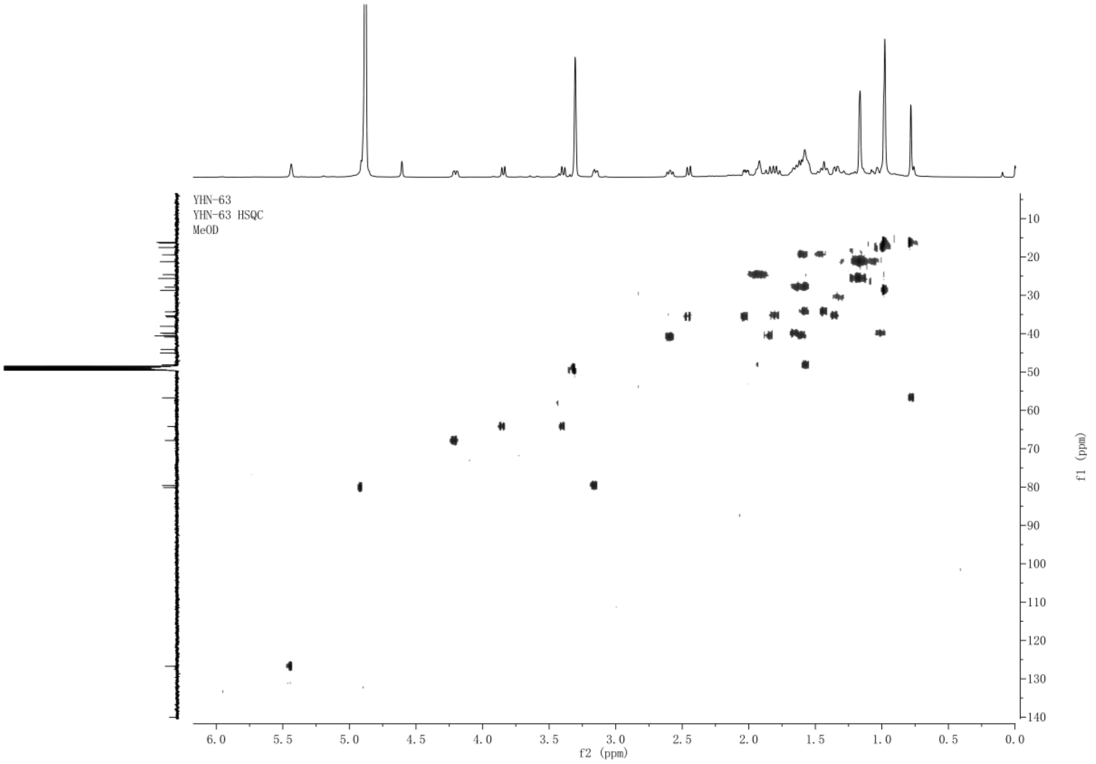

Figure S19. HSQC spectrum of **3** in CD_3OD

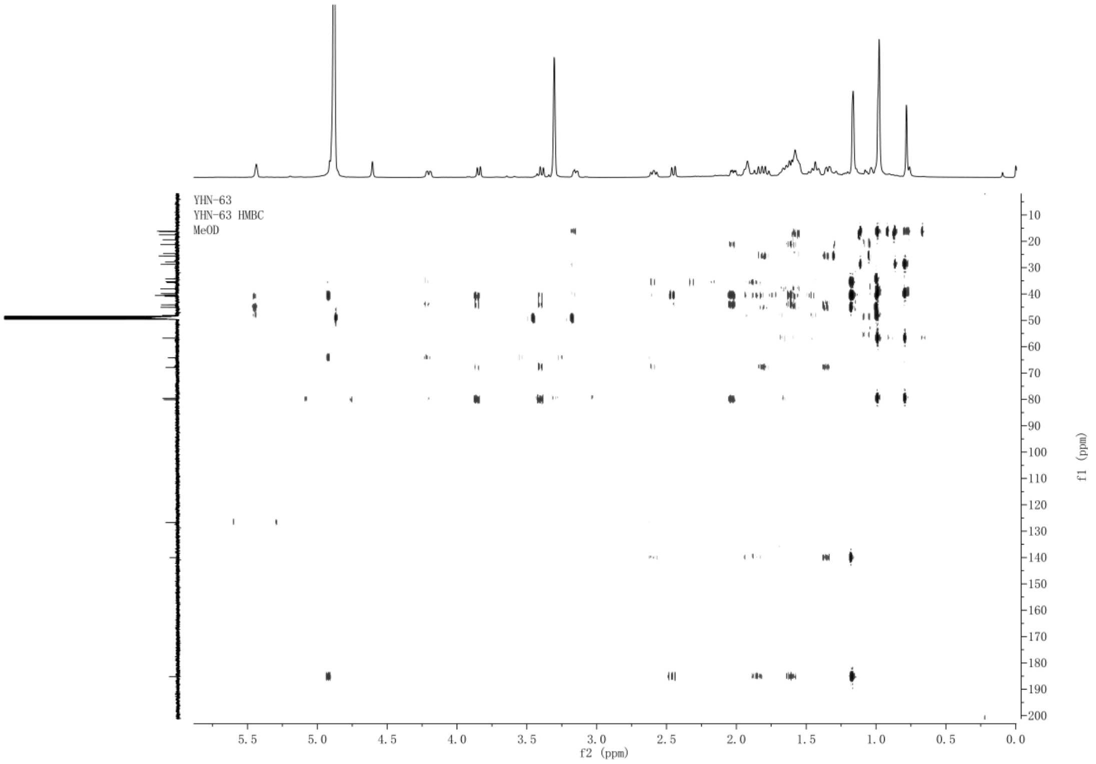

Figure S20. HMBC spectrum of **3** in CD_3OD

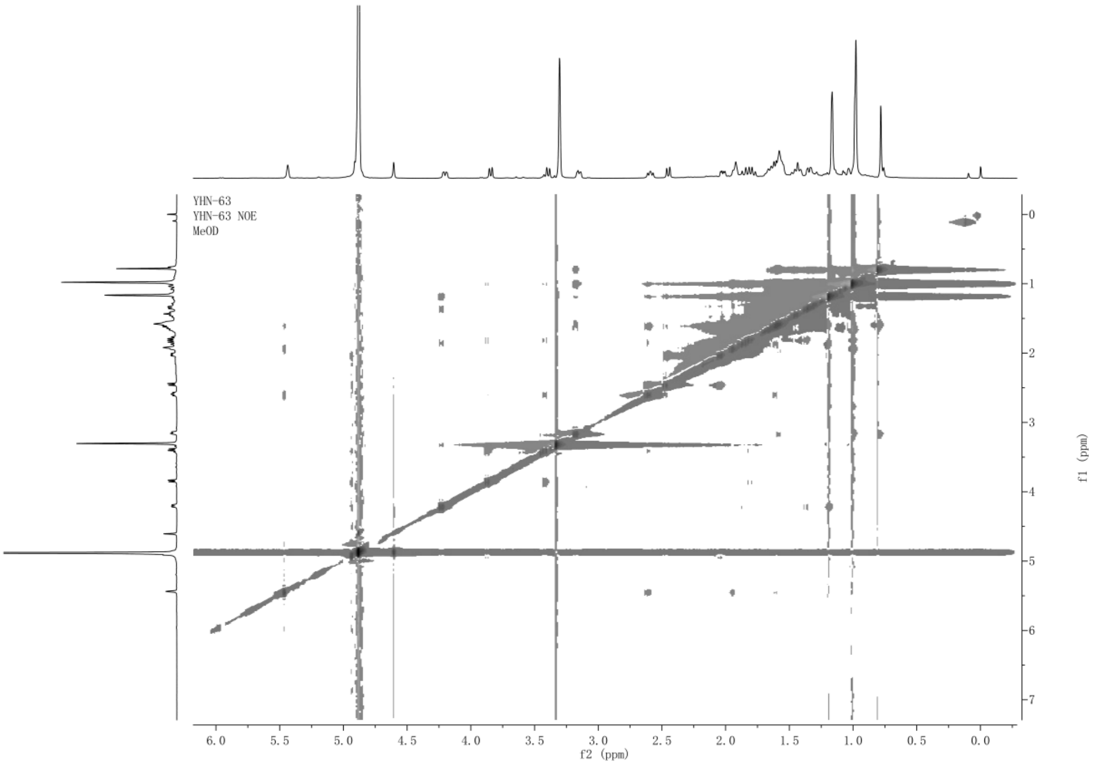

Figure S21. NOESY spectrum of **3** in CD_3OD
